# Hepatic stellate cells secrete Ccl5 to induce hepatocyte steatosis

**DOI:** 10.1038/s41598-018-25699-9

**Published:** 2018-05-14

**Authors:** Byeong-Moo Kim, Ahmed Maher Abdelfattah, Robin Vasan, Bryan C. Fuchs, Michael Y. Choi

**Affiliations:** 1 Department of Medicine, Gastrointestinal Unit, Massachusetts General Hospital and Harvard Medical School, Boston, MA 02114 USA; 2 Department of Surgery, Massachusetts General Hospital Cancer Center and Harvard Medical School, Boston, MA 02114 USA; 3 Division of Surgical Oncology, Massachusetts General Hospital Cancer Center and Harvard Medical School, Boston, MA 02114 USA; 4 Harvard Stem Cell Institute, Cambridge, MA 02138 USA

**Keywords:** Mechanisms of disease, Non-alcoholic fatty liver disease

## Abstract

Non-alcoholic fatty liver disease (NAFLD) encompasses a wide spectrum of disease severity, starting from pure steatosis, leading to fatty inflammation labeled as non-alcoholic steatohepatitis (NASH), and finally fibrosis leading to cirrhosis. Activated hepatic stellate cells (HSCs) are known to contribute to fibrosis, but less is known about their function during NAFLD’s early stages prior to fibrosis. We developed an *ex vivo* assay that cocultures primary HSCs from mouse models of liver disease with healthy hepatocytes to study their interaction. Our data indicate that chemokine Ccl5 is one of the HSC-secreted mediators in early NASH in humans and in mice fed with choline-deficient, L-amino acid defined, high fat diet. Furthermore, Ccl5 directly induces steatosis and pro-inflammatory factors in healthy hepatocytes through the receptor Ccr5. Although Ccl5 is already known to be secreted by many liver cell types including HSCs and its pro-fibrotic role well characterized, its pro-steatotic action has not been recognized until now. Similarly, the function of HSCs in fibrogenesis is widely accepted, but their pro-steatotic role has been unclear. Our result suggests that in early NASH, HSCs secrete Ccl5 which contributes to a broad array of mechanisms by which hepatic steatosis and inflammation are achieved.

## Introduction

Hepatic stellate cells (HSCs) are considered to play a central role in hepatic fibrogenesis^1–4^. Since fibrosis is the common pathway that ultimately leads to cirrhotic organ failure or hepatocellular carcinoma that occurs in progressive liver diseases, understanding the biology of HSCs is pivotal for all liver diseases that can lead to hepatic fibrosis. This paradigm is particularly important in non-alcoholic fatty liver disease (NAFLD) which has become the most significant hepatic illness in the world. Just in the United States NAFLD affects over 64 million people by a recent estimate^5^, and it will soon become the most common cause of liver transplant and hepatocellular carcinoma^6,7^. Although several drugs are undergoing clinical trials there are still no well-established pharmacologic agents to treat NAFLD, and the main mode of management is through reducing risk factors such as obesity, hyperlipidemia, and diabetes^8^. Hence, elucidating the HSC pathobiology in NAFLD is crucial to identify novel treatment options.

Although fibrosis that leads to end-stage organ failure or cancer is an important feature of NAFLD, the disease does not start with fibrosis. Instead, NAFLD encompasses a wide spectrum of disease severity, starting from pure steatosis, leading to fatty inflammation labeled as non-alcoholic steatohepatitis (NASH), and finally fibrosis leading to cirrhosis^9,10^. HSCs are known to contribute to fibrosis, but much less is known about their function during NAFLD’s earlier stages, in steatosis and inflammation without fibrosis. One of the difficulties in studying HSCs in the context of other cell types during the early stages of NAFLD is that they are relatively hard to identify *in vivo* to study their role and that insightful *in vitro* assays that model HSC’s interaction with other cell types are difficult to develop. In response, we have devised an *ex vivo* assay that cocultures healthy hepatocytes with primary HSCs from mouse models of steatohepatitis to study their interaction. The assay greatly enhances our ability to define HSC function and the mechanism of its action during fatty inflammation of the liver by identifying HSC-secreted mediators that have profound effects on nearby hepatocytes.

In healthy humans and mice, HSCs are quiescent, residing near sinusoids in the space of Disse. However, once the liver is afflicted with a chronic illness such as NAFLD/NASH, quiescent HSCs become transdifferentiated to give rise to myofibroblasts^11^. These activated HSCs, termed myofibroblasts, are known to express cytokines, chemokines, extracellular matrix proteins, and other genes that contribute to hepatic fibrogenesis^12^. Our data indicate that chemokine (C-C motif) ligand 5 (Ccl5, a.k.a. Rantes) is one of the HSC-secreted mediators in NASH that directly induce steatosis and pro-inflammatory factors in initially healthy hepatocytes. Another group already demonstrated that human HSCs express CCL5 when challenged with TNF-alpha, IL-1beta, or CD40L *in vitro*^13^, but our work suggests for the first time that HSCs express Ccl5 in early NASH to directly induce steatosis and upregulation of pro-inflammatory factors in hepatocytes. Although Ccl5 is also expressed by other liver cell types^14–17^ and is better known for causing hepatic fibrosis through the recruitment of immune cells^14,15^, its pro-steatotic action in early NAFLD/NASH has not been recognized until now. Similarly, the essential function of HSCs in hepatic fibrogenesis is well accepted, but their pro-steatotic role, however minor, in directly influencing hepatocytes in early NAFLD/NASH has not been clear. Using a mouse model of steatohepatitis, our result suggests that HSCs have a function in inducing steatosis and inflammation, not just fibrosis.

## Results

### Mice fed a choline-deficient, L-amino acid-defined, high fat diet for three weeks develop steatohepatitis

**Figure 1:**
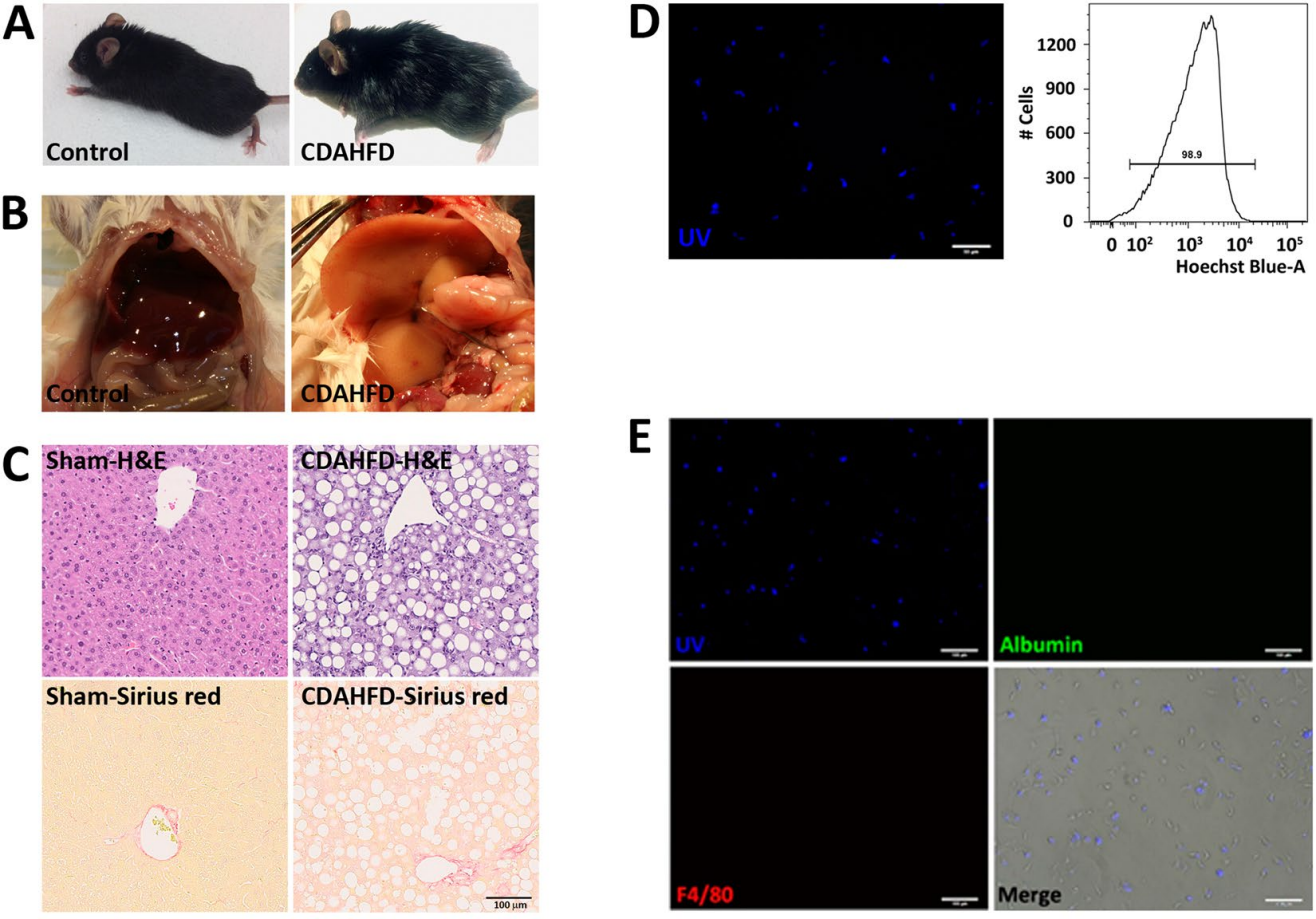
Mouse model of steatohepatitis using choline-deficient, L-amino acid-defined, high fat diet. (**A**–**C**) After three weeks of CDAHFD, mice were both grossly and microscopically affected with steatohepatitis that resembles early NASH. (**D**) Hepatic stellate cells harvested from CDAHFD fed mice retained retinoid positive lipid droplets that fluoresce with ultraviolet light. Nearly 99% of the isolated hepatic stellate cell population fluoresced with ultraviolet light based on FACS analysis. (**E**) The harvested hepatic stellate cell population did not express albumin or F4/80 based on immunofluorescence. CDAHFD, L-amino acid-defined, high fat diet; NASH, non-alcoholic steatohepatitis; UV, ultraviolet.

NAFLD starts as simple fatty liver that can progress to steatohepatitis, fibrosis, and ultimately cirrhosis and hepatocellular carcinoma^18^. Although HSCs are known to have a critical function in the development of hepatic fibrosis by secreting extracellular matrix proteins and expressing other pro-fibrotic genes^1–3^, less is known about their action during the initial, fatty liver and the early inflammatory phases of NASH. To study their function during this early stage of the disease, we isolated HSCs from a mouse model of steatohepatitis relying on choline-deficient, L-amino acid-defined, high fat diet (CDAHFD). These mice normally develop severe steatosis with moderate inflammation but no fibrosis within 3 weeks, mild fibrosis within 6 weeks, moderate fibrosis in 9 weeks, and moderate to severe fibrosis in 12 weeks^19^. Thus, mice fed with CDAHFD for three weeks have fatty and inflamed liver that resembles the histology of early NASH prior to fibrosis. Grossly, at three weeks, their skin became oily, evidenced by the greasy coat that sticks together in clumps (Fig. 1A). Internally, their livers were lighter in color, demonstrating hepatic fat (Fig. 1B). Microscopically, they showed several large and small lipid droplets in the cytoplasm of hepatocytes, confirming moderate to severe steatosis with moderate inflammation but no fibrosis (Fig. 1C). To study the role of HSCs at this stage of liver disease, we isolated them by optimizing and improving on an established protocol^20^. Each 8-week-old female C57BL/6 mouse fed with either normal diet or CDAHFD for three weeks yielded 0.5–1 million HSCs. This population of cells was 98.9% pure for positive fluorescence with ultraviolet light (Fig. 1D), a signal specific for retinoid filled lipid droplets present in quiescent HSCs. Furthermore, absence of cells expressing albumin or F4/80 in this population confirmed almost no contamination of hepatocytes or macrophages, respectively (Fig. 1E).

### Primary HSCs isolated from mice with non-fibrotic steatohepatitis more quickly become activated in culture

**Figure 2:**
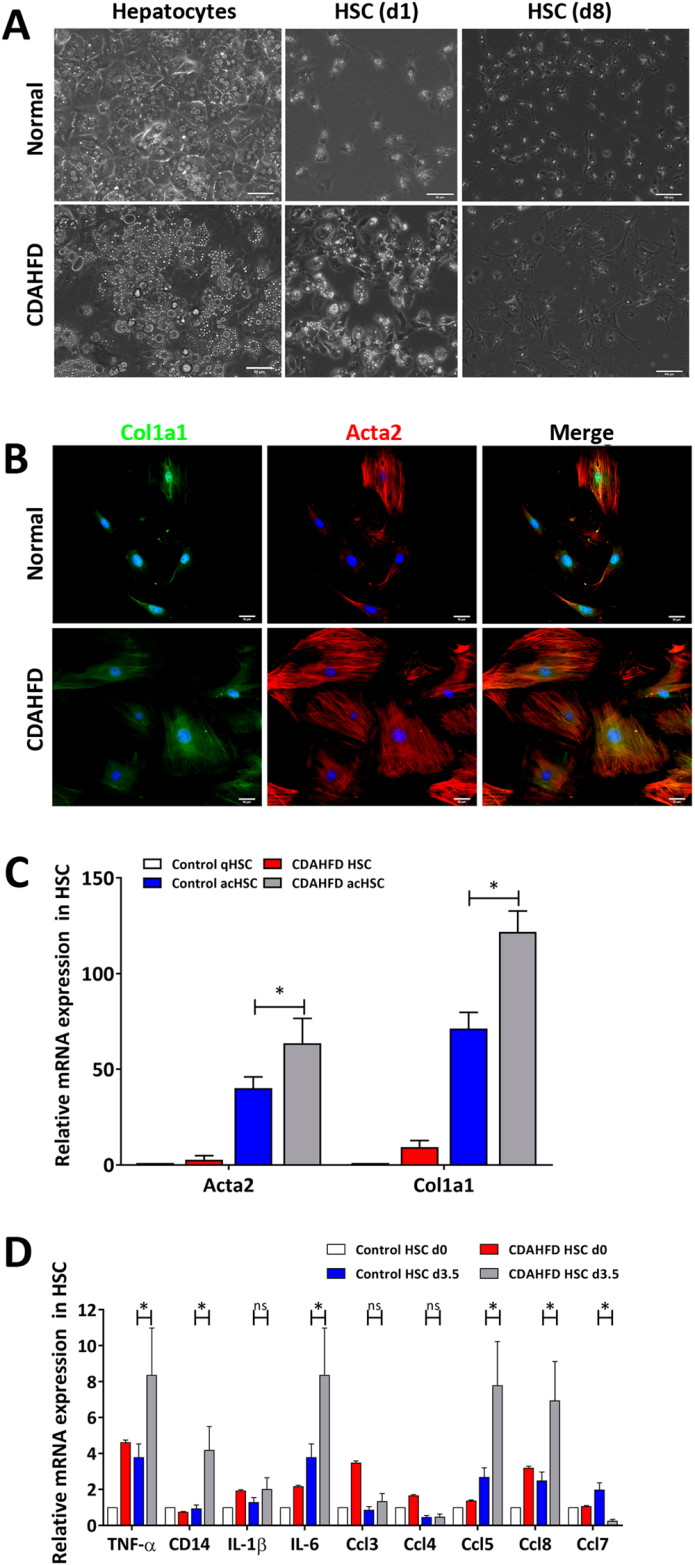
Hepatic stellate cells from mice with steatohepatitis induced by CDAHFD are primed to become activated. (**A**) Hepatocytes from mice with CDAHFD induced steatohepatitis were steatotic while hepatic stellate cells were only slightly larger in size compared to those from healthy mice. After eight days of culture, hepatic stellate cells from early NASH became much larger in size compared to control stellate cells. (**B**) Hepatic stellate cells from mice with CDAHFD induced steatohepatitis more quickly increased in size and in expression of activation markers Colla1 and Acta2, visualized here just three days after the initial cell isolation. (**C**) Hepatic stellate cells from mice with CDAHFD induced steatohepatitis maintained higher expression of Col1a1 and Acta2 than those from control mice even after both populations were fully activated on plastic dish. Expression levels measured with qPCR. (**D**) Hepatic stellate cells from mice with CDAHFD induced steatohepatitis had elevated expression of several pro-inflammatory cytokines compared to those from control mice. Expression levels measured with qPCR. All data are presented as mean +/− SD (*P < 0.05). NASH, non-alcoholic steatohepatitis; CDAHFD, choline-deficient L-amino acid defined high fat diet; HSC, hepatic stellate cell; qHSC, quiescent hepatic stellate cell; acHSC, activated hepatic stellate cell; Col1a1, alpha-1 type I collagen; Acta2, alpha smooth muscle actin; ns, not significant.

Quiescent HSCs are known to become activated spontaneously to myofibroblasts if they are cultured on plastic dish for 7–10 days^21,22^. This phenomenon is likely driven by the high degree of stiffness inherent to plastic which acts as the activating signal to HSCs, and this same mechanism of action may also take place in the stiffening hepatic tissue as fibrosis progresses which itself becomes one of many activating signals *in vivo*^23^. HSCs harvested from liver with steatohepatitis without fibrosis induced by three weeks of CDAHFD morphologically resembled quiescent HSCs from healthy mice, retaining UV-fluorescing lipid droplets, but they were slightly larger in size (Figs 1D and 2A). More significantly, they were functionally distinct. Their morphologic transdifferentiation to activated HSCs occurred more quickly in culture, requiring only 3–6 days, instead of 7–10 days. As opposed to the usual seven days, by the third day in culture, HSCs isolated from CDAHFD induced non-fibrotic steatohepatitis increased in size with the spread morphology (Fig. 2B). Furthermore, by day 3.5 in culture, these HSCs had heightened expression of activation markers alpha smooth muscle actin (Acta2) and alpha-1 type I collagen (Col1a1) covering the expanded cytosol (Fig. 2B). This elevated expression of activation markers was maintained even after HSCs became fully activated on plastic dish (Fig. 2C). Finally, we analyzed the expression of several cytokines and chemokines involved in inflammation and recruitment of immune cells, and HSCs from steatohepatitic liver showed significantly higher expression of tumor necrosis factor-alpha (*Tnf*), cluster of differentiation 14 (*Cd14*), interleukin 6 (*Il-6*), and chemokine (C-C motif) ligand 5 (*Ccl5*) and 8 (*Ccl8*) by day 3.5 in culture compared to HSCs from healthy mice (Fig. 2D). These results indicate that although HSCs isolated from the liver with CDAHFD induced steatohepatitis resembling early NASH have a quiescent morphology with retinoid positive lipid droplets, they are intrinsically different from quiescent HSCs and primed to become fully activated.

### Primary HSCs isolated from non-fibrotic steatohepatitic liver induce steatosis and inflammation in healthy hepatocytes

**Figure 3:**
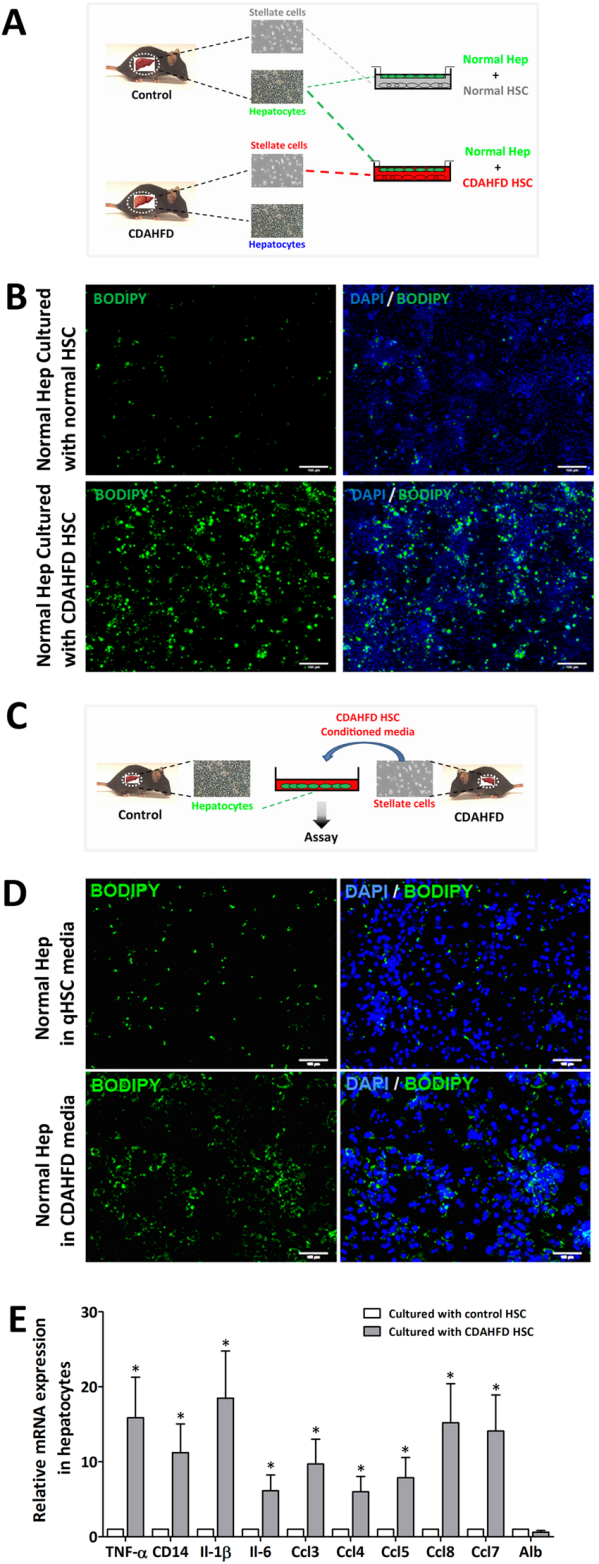
Hepatic stellate cells isolated from mice with steatohepatitis induce steatosis and inflammation in cocultured hepatocytes. (**A**) Diagram of the experiment showing coculture of healthy hepatocytes with hepatic stellate cells from either control mice or from mice with CDAHFD induced steatohepatitis. (**B**) Top row: Healthy hepatocytes cocultured with HSCs from control mice showed very few lipid droplets stained with Bodipy. Bottom row: Initially healthy hepatocytes cocultured with HSCs from mice with CDAHFD induced steatohepatitis developed significantly more lipid droplets. (**C**) Induction of fatty accumulation in hepatocytes can be achieved when the conditioned media from HSCs from mice with CDAHFD induced steatohepatitis is applied to healthy hepatocytes. (**D**) Top row: When quiescent HSC conditioned media was applied to healthy hepatocytes, steatosis was not induced. Bottom row: Conditioned media of HSCs from mice with CDAHFD induced steatohepatitis induced steatosis in initially healthy hepatocytes. Lipid droplets stained with Bodipy. (**E**) Initially healthy hepatocytes cocultured with HSCs from mice with CDAHFD induced steatohepatitis expressed higher levels of several inflammatory cytokines and chemoattractants than when they were cocultured with control HSCs, measured with qPCR. Data are presented as mean +/− SD (*P < 0.05). NASH, non-alcoholic steatohepatitis; CDAHFD, choline-deficient L-amino acid defined high fat diet; Hep, hepatocyte; HSC, hepatic stellate cell.

Since HSCs from steatohepatitic liver are more susceptible to become activated, phenotypically distinct from quiescent HSCs, we tested their ability to influence nearby hepatocytes. When we cocultured primed HSCs from three weeks of CDAHFD with hepatocytes isolated from mice eating standard diet, initially healthy hepatocytes became steatotic with numerous lipid droplets within three days (Fig. 3A,B). This result was obtained while HSC-hepatocyte coculture was performed using a Transwell that physically separates the two cell types while secreted proteins and small molecules can freely traverse this barrier, suggesting that HSCs are inducing hepatocyte steatosis not through cell-cell contact but by secreting mediators. We verified this phenomenon when steatosis was induced in healthy hepatocytes by applying the conditioned media of primed HSCs harvested from mice challenged with three weeks of CDAHFD (Fig. 3C,D). Finally, we evaluated the expression levels of various pro-inflammatory cytokines and chemokines in steatotic hepatocytes cocultured with primed HSCs. The result shows that these hepatocytes were not only steatotic but also expressed many pro-inflammatory genes that further activate HSCs and attract immune cells (Fig. 3E).

### Hepatocyte steatosis is induced by Ccl5 secreted from HSCs

**Figure 4:**
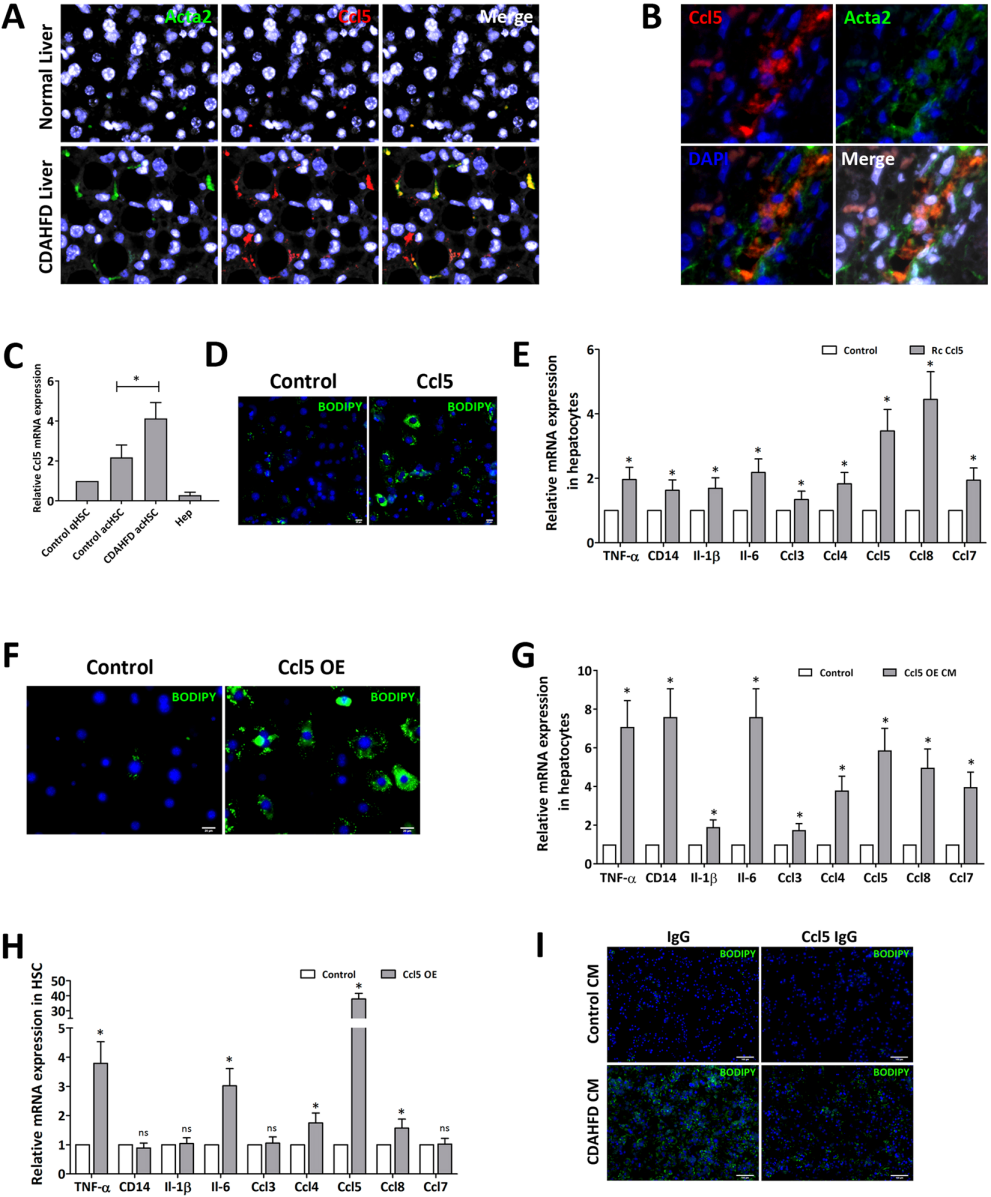
Ccl5 is secreted by hepatic stellate cells isolated from mice with CDAHFD induced steatohepatitis and induces hepatocyte steatosis. (**A**,**B**) Expression of Ccl5 and Acta2 co-localized to hepatic stellate cells in both mouse steatohepatitis and human NASH assessed by immunofluorescence (7 out of 11 human samples tested positive). (**C**) Hepatic stellate cells isolated from mice with steatohepatitis continued to express Ccl5 at a greater level than initially healthy hepatic stellate cells, even after further activation on dish, measured with qPCR. (**D**) Recombinant Ccl5 protein induced steatosis in freshly isolated primary mouse hepatocytes, observed with Bodipy staining. (**E**) Hepatocytes that became steatotic with recombinant Ccl5 upregulated pro-inflammatory cytokines and chemokines, measured with qPCR. (**F**,**G**) The conditioned media from Ccl5 overexpressing hepatic stellate cells induced steatosis, detected with Bodipy stain, and caused increased expression of several inflammatory cytokines and chemoattractants in primary hepatocytes, measured with qPCR. (**H**) Hepatic stellate cells overexpressing Ccl5 also had increased expression of other pro-inflammatory mediators besides Ccl5, measured with qPCR. All data are presented as mean +/− SD (*P < 0.05). (**I**) Neutralizing Ccl5 in HSC conditioned media with a blocking antibody reduced steatosis in hepatocytes treated with the media. NASH, non-alcoholic steatohepatitis; HSC, hepatic stellate cell; CDAHFD, choline-deficient L-amino acid defined high fat diet; Hep, hepatocyte; qHSC, quiescent hepatic stellate cell; acHSC, activated hepatic stellate cell; Rc, recombinant; OE, overexpression; CM, conditioned media; ns, not significant.

Since hepatocyte steatosis did not require cell-cell contact with HSCs originating from steatohepatitic liver, steatosis inducing signals are likely transmitted through secreted mediators in a paracrine manner. To identify the secreted factors responsible for inducing steatosis in nearby hepatocytes, we performed a cytokine array blot of the conditioned media from HSCs in culture. One of the highly up-secreted proteins was Ccl5 (Supp. Fig. 1), which was already demonstrated to be upregulated at the transcriptional level (Fig. 2D), and enzyme-linked immunosorbent assay (ELISA) confirmed the up-secretion of Ccl5 from these HSCs (Supp. Fig. 4). Finally, the upregulation of Ccl5 in HSCs from CDAHFD induced steatohepatitis in mice and early NASH in humans was further verified by immunofluorescence which showed co-localization of Ccl5 with Acta2, an activated HSC marker in the liver (Fig. 4A,B). Moreover, these HSCs continued to express Ccl5 at a greater level than initially normal HSCs, even after further activation on dish (Fig. 4C). Although Ccl5 is a relatively well-investigated chemokine, known to promote hepatic fibrosis, its possible pro-steatotic effect has not been investigated^14,15^. To test this hypothesis, we investigated whether Ccl5 directly causes steatosis in hepatocytes or is simply an upregulated gene with an unrelated function. First, we applied recombinant Ccl5 protein at various concentrations to nontransformed mouse hepatocyte cell line AML12 in culture. Indeed, purified Ccl5 protein induced steatosis in AML12 cells starting at 1 ng/ml, but more robustly with 50 ng/ml and 100 ng/ml (Supp. Fig. 2). Ccl5 also caused steatosis in primary mouse hepatocytes at these concentrations, seen with Bodipy stain (Fig. 4D). Furthermore, these hepatocytes that became steatotic with recombinant Ccl5 upregulated pro-inflammatory cytokines and chemokines of their own (Fig. 4E), demonstrating Ccl5’s pro-steatotic and pro-inflammatory effects. Interestingly, applying recombinant Ccl5 to hepatocytes caused those hepatocytes to upregulate Ccl5 themselves in a feed-forward manner. To further understand this HSC-hepatocyte interaction involving Ccl5, we cloned the full length Ccl5 gene and overexpressed it in HSCs (Supp. Fig. 3). We collected the conditioned media from these Ccl5 expressing HSCs, and applied it on healthy hepatocytes. As expected, these hepatocytes also formed lipid droplets, detected by Bodipy stain (Fig. 4F). Furthermore, these same hepatocytes increased expression of various pro-inflammatory cytokines and chemokines but in greater folds than when recombinant Ccl5 was directly applied (Fig. 4G). To investigate the reason for this greater induction of pro-inflammatory cytokines and chemokines in hepatocytes by Ccl5 overexpressing HSCs, we checked the expression levels of other cytokines and chemokines besides Ccl5 in these cells. It turns out, the HSCs overexpressing Ccl5 also had increased expression of other pro-inflammatory mediators besides Ccl5, suggesting that Ccl5 induces either an autocrine signaling or purely an intracellular signaling cascade that leads to upregulation of genes such as *Tnf* and *Il-6* (Fig. 4H). Pro-inflammatory cytokines such as Tnf, Il-6, and unidentified factors upregulated by Ccl5 in HSCs are likely being secreted and further inducing certain pro-inflammatory gene expression in nearby hepatocytes. Lastly, the pro-steatotic effect of Ccl5 secreted by HSCs was further evidenced when a Ccl5 neutralizing antibody applied to CDAHFD-HSC conditioned media attenuated its induction of hepatocyte steatosis (Fig. 4I, Supp. Fig. 5). We also confirmed that the source of Ccl5 is HSCs, not hepatocytes, in these assays by demonstrating the lack of Ccl5 immunofluorescence signal from hepatocyte culture (Supp. Fig. 6).

### Blocking the action of Ccl5 using an inhibitor of Ccr5 decreases hepatocyte steatosis *in vitro*

**Figure 5:**
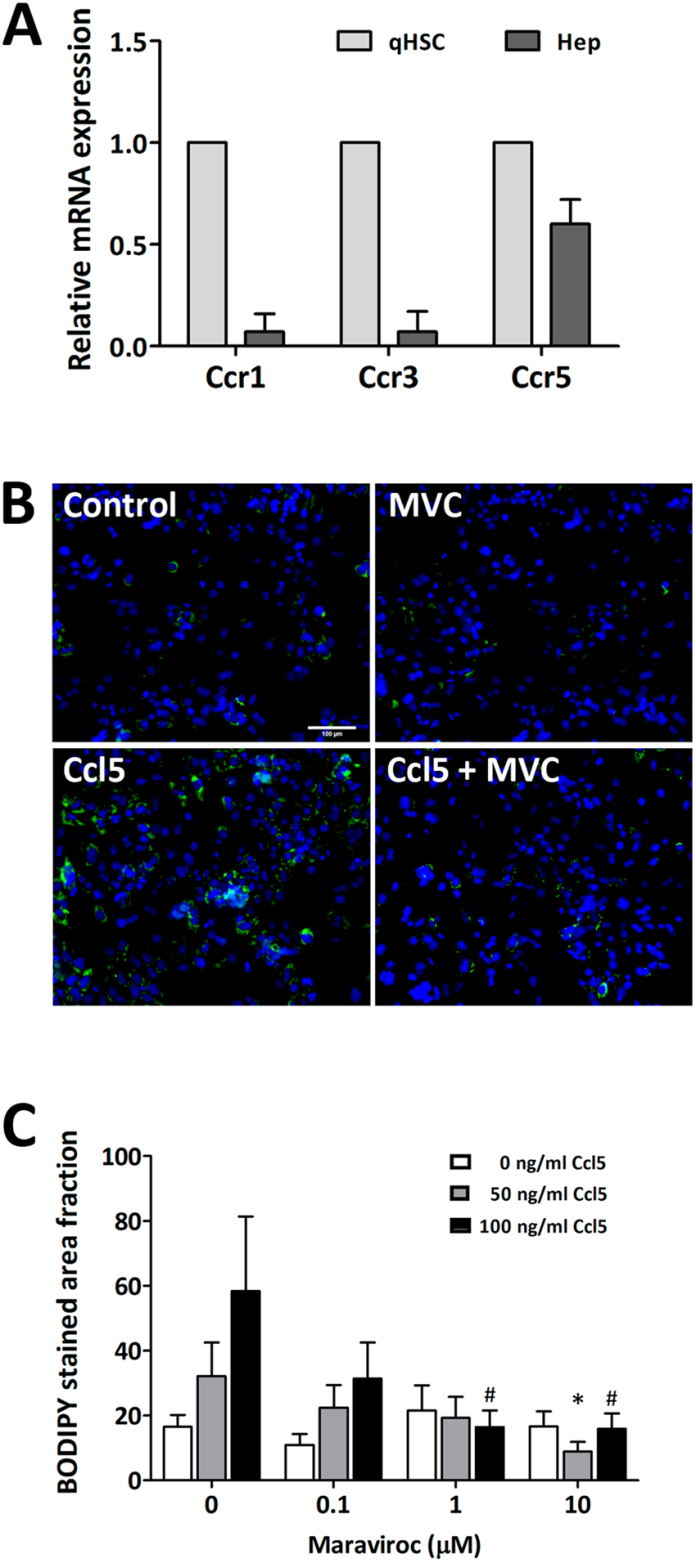
Ccr5 specific inhibitor Maraviroc attenuates pro-steatotic effect of Ccl5 on hepatocytes. (**A**) Primary hepatocytes express Ccr5 at a level significantly higher than Ccr1 or Ccr3, measured with qPCR. (**B**,**C**) Ccr5 specific inhibitor Maraviroc attenuated pro-steatotic effect of Ccl5 on primary hepatocytes, the level of steatosis quantified with Bodipy stained area fraction. Data are presented as mean +/− SD (*P < 0.05 for comparisons among cells receiving 50 ng/ml of Ccl5; ^#^P < 0.05 for comparisons among cells receiving 100 ng/ml of Ccl5). qHSC, quiescent hepatic stellate cell; Hep, hepatocyte; MVC, Maraviroc.

Ccl5 is known to interact with the membrane C-C chemokine receptors including type 1 (Ccr1), type 3 (Ccr3), and type 5 (Ccr5)^14,15,24^. To determine expression levels of *Ccr1*, *Ccr3*, and *Ccr5*, we performed quantitative PCR (qPCR)of these three genes in primary mouse hepatocytes. The result showed that hepatocytes express *Ccr5* significantly more than *Ccr1* or *Ccr3* (Fig. 5A). To test whether Ccl5 expressed by HSCs signal through Ccr5 on hepatocytes to induce steatosis and to upregulate pro-inflammatory cytokines, we applied both recombinant Ccl5 and the Ccr5-specific inhibitor Maraviroc on hepatocytes. As suspected, pro-steatotic effect of Ccl5 was attenuated with Maraviroc (Fig. 5B,C).

## Discussion

Our study accomplishes two goals that should further facilitate the study of HSC biology in the setting of NASH. First, knowing that studying HSC’s interaction with nearby hepatocytes in their microenvironment during liver diseases would be facilitated by having a reliable *ex vivo* system that uses primary cells, we developed our coculture system using a mouse model of steatohepatitis histologically resembling early NASH. Using primary HSCs was important because malignant or immortalized cell lines often do not reflect the molecular physiology and the measurable behavior of primary cells^25^. Also, the mouse as a species provides a vast array of genetic, diet, chemical, and anatomic models of liver diseases. Although there is no perfect animal model that completely recapitulates human NASH, we chose to utilize the CDAHFD model because it establishes severe steatosis with moderate inflammation in three weeks which eventually leads to fibrosis in subsequent weeks, the stepwise progression seen in humans^19^. Our coculture system allowed us to better define the function and its mechanism of HSCs in affecting nearby hepatocytes during steatohepatitis development. Several points should be noted here. Although we studied the function of diseased HSC’s influence on healthy hepatocyte, the system also can be applied to investigate diseased hepatocyte’s action on healthy HSC. In fact, other important cell types such as macrophages and sinusoidal endothelial cells could potentially be used as long as a highly pure population can be isolated and cultured. Moreover, although we have not tried, it is possible to coculture three or more cell types together to study the interactions among them. The cell type directly harvested from a diseased liver should more accurately reflect its diseased phenotype, which is the strength of our *ex vivo* system. However, it must be emphasized that terminally differentiated primary cells often cannot be maintained in culture for a prolonged period without changes in their phenotype. This is the reason why our coculture assays in this study did not extend beyond three days in culture. We believe that any terminally differentiated primary cells cultured *ex vivo* for more than 5–7 days lose their original phenotype. Furthermore, the insight we gain from studying primary cells in a diseased context is only as good as the animal model. Nevertheless, our coculture system is a useful tool to better define the role of hepatic cell types and their interactions in the setting of various liver diseases.

Second, using the coculture assay we developed, we wanted to better understand the function of HSCs in a mouse model of non-fibrotic steatohepatitis that may simulate early NASH prior to the onset of fibrosis. Our data demonstrate that HSCs directly induce steatosis in healthy hepatocytes by secreting mediators that act in a paracrine fashion. One of those mediators that have a direct steatotic effect on hepatocytes is Ccl5 which seems to exert its effect through Ccr5. This unexpected result suggests that HSCs have pathophysiologic functions beyond laying down excessive amount of extracellular matrix to cause fibrosis during the mid to late stages of NASH. We should caution, however, that this new finding does not necessarily indicate that the pro-steatotic role of HSCs or Ccl5 during the early stage NASH is the major mechanism by which liver becomes steatotic. For example, it is known that Ccl5 is secreted by other cell types such as macrophages, platelets, endothelial, T cells, and hepatocytes^14–17^. Hence, hepatocytes likely become steatotic through multiple molecular signals and cell types, and the proportion of the phenotype contributed by HSCs through Ccl5 may be minor. Interestingly, supporting the mild pro-steatotic function of Ccl5, Ccl5 knockout mice demonstrate a trend toward lower hepatic triglyceride levels compare to their wildtype littermates^15^. Finally, although hepatocytes are generally considered the main driver of inflammation that signals to other cell types including HSCs, our result suggests that a significant amount of pro-inflammatory signals may reciprocate back to hepatocytes from HSCs to create a vicious circle of reinforcing inflammatory signals^12^. Although not directly tested in this study, such feed-forward escalation in hepatic inflammation likely involves other cell types as well.

In summary, our coculture assay using primary cells from the liver is a versatile method by which the interaction between isolatable cell types from any mouse model of liver disease can be researched. The system attempts to faithfully recreate the microenvironment of cells within the liver by culturing freshly harvested primary cells for a short duration, lasting no more than 5–7 days. We applied the method to coculture diseased HSCs from steatohepatitic liver with healthy hepatocytes for three days, and discovered that HSCs can directly induce hepatocytes to become steatotic by secreting the chemokine Ccl5. Although this result does not preclude the possibility of other secreted factors or cell types causing similar phenotype, the data reveals a novel function of Ccl5 and HSCs. Our coculture system has the potential to uncover other therapeutically relevant functions of HSCs and other liver cell types, which is urgently needed given the rising incidence of NASH and the lack of FDA-approved therapies.

## Methods

### Animals

All experiments were performed in accordance with the National Institutes of Health’s Guide for the Care of Use of Laboratory Animals and approved by the Institution Animal Care and Use Committee and the Subcommittee on Research Animal Care at Massachusetts General Hospital. Eight week old C57BL/6 female mice were purchased from Charles River Laboratories, Wilmington, MA. The animals were maintained under a 12-hour light-dark cycle with free access to standard laboratory chow diet or a choline-deficient, L-amino acid-defined, high-fat diet (CDAHFD) consisting of 60% kcal fat and 0.1% methionine by weight in order to induce steatohepatitis^19^.

### Hepatic stellate cells and hepatocyte isolation

Hepatocytes were isolated from C57BL/6 mice fed the control diet and hepatic stellate cells isolated from mice fed the control diet or the CDAHFD by enzymatic digestion and Percoll density gradient centrifugation with modifications. The portal vein was perfused *in situ* with 30 mL of HBSS (without Ca2^+^ and Mg2^+^) and 30 mL of 0.05% collagenase B (Roche Diagnostics, Indianapolis, IN, USA), respectively, at 37 °C with a flow rate of 6 ml/min. After perfusion, the partially digested liver was excised and incubated with 20 mL of 0.05% collagenase and DNase I 10 μg/mL, (Roche Diagnostics, Indianapolis, IN), at 37 °C for 30 minutes. The tissue was passed through a 70 µm nylon mesh to remove undigested materials and suspended in washing buffer (PBS containing DNase I). Hepatocytes were separated from the non-parenchymal cells and debris by centrifugation at 4 °C in the following sequence: twice for 5 minutes at 50 g, and twice again for 5 minutes at 20 g. The supernatant was collected for stellate cell isolation and the hepatocytes present in the pellet were re-suspended in DMEM. Primary mouse HSCs were purified from the remainder of non-parenchymal cells. Cells were centrifuged at 635 g for 10 minutes and resuspended in washing buffer followed by pass through a 70 µm nylon mesh. The pellets were resuspended in 10 ml of 35% Percoll (GE Healthcare, Pittsburgh, PA, USA) with an overlay of 1 ml PBS. After centrifugation at 1130 g for 30 minutes, HSCs are in the layer located between the PBS and 35% Percoll. Cells were counted using a hemocytometer (Neubauer chamber) and 0.4% trypan blue (Sigma-Aldrich, St. Louis, MO, USA). The purity was determined by UV sorting with BD FACS Aria II SORP Cell Sorter (BD Biosciences, San Jose, CA, USA).

### Cell culture

Isolated hepatocytes were re-suspended in DMEM medium containing 10% FBS, plated onto collagen-coated six-well plates at a density of 5 × 10^5^ cells/well in 1.5 ml culture medium, and cultured for 4 hours. The medium was then changed into serum-free medium and the cells were cocultured with hepatic stellate cells and loaded onto cell-culture inserts of 3 µm pore size (Corning, Corning, NY, USA). For recombinant Rantes treatment, various concentration of mouse recombinant Rantes (R & D Systems, Minneapolis, MN, USA) were added to cells for 3 days. To test inhibition of Rantes, cells were preincubated with recombinant Ccl5 for 12 hours before addition of Maraviroc (MVC, TOCRIS, Bristol, UK).

### Human tissues

Eleven patients who underwent liver biopsies at the Massachusetts General Hospital and were diagnosed with NASH were identified from the pathology database. The archived tissues embedded in paraffin blocks were processed for immunofluorescence analysis. This study was approved by the Partners Human Research Committee.

### BODIPY staining of lipid droplets

Hepatocytes co-cultured with stellate cells or conditioned media were washed with PBS, fixed with 4% formaldehyde for 15 minutes, and stained with BODIPY (1 µg/ml, Invitrogen, Carlsbad, CA, USA) for 15 min at room temperature. Cells were then washed 3 times with PBS and stained with DAPI.

### Quantitative Real-Time PCR

Total RNA was extracted with TRIzol reagent (Invitrogen, Carlsbad, USA) and reverse transcribed into cDNA using a Superscript IV (Invitrogen, Carlsbad, USA). SYBR-Green real-time RT-PCR was performed with listed primers (Supp. Table 1) and GAPDH mRNA was used to normalize RNA inputs.

### Immunofluorescence

Cells were fixed with 4% paraformaldehyde for 10 minutes at room temperature and permeabilized with 0.3% Triton X-100 in phosphate-buffered saline (PBS) for 20 minutes. Nonspecific immunostaining was prevented by 30 minutes’ incubation of the cells in PBS solution containing 20% normal goat serum (DakoCytomation, Glostrup, Denmark) at room temperature. Cells were then incubated overnight at 4 °C with primary antibodies as follows: Ccl5 (R & D Systems, Minneapolis, MN, USA), albumin (Bethyl Lab, Montgomery, TX, USA; Abcam, Cambridge, MA) and alpha smooth muscle actin (BioGenex, Fremont, CA, USA; Abcam, Cambridge, MA, USA), F4/80 (Bio-Rad, Hercules, CA, USA), Col1a1 (ThermoFisher, Waltham, MA, USA). Alexa Fluor 488, 555 goat anti-mouse IgG (H + L) (Molecular probes, Eugene, USA) were used as secondary antibodies. Nuclei were revealed by 3 minutes of staining with the nuclear dye 4′, 6-diamidino-2-phenylindole (DAPI) (Invitrogen, Carlsbad, CA, USA). After 3 washes, cells were mounted with antifading solution (Vector Laboratories, Burlingame, CA, USA) and examined under fluorescence microscope (BX51) (Olympus, Japan).

### Fluorescence-Activated Cell Sorting

Cell sorting was done using a BD FACS Aria II SORP Cell Sorter (BD Biosciences, Franklin Lakes, NJ, USA). The pellet was resolved in 4 °C Hank’s complete and filtered using 40 μm nylon gaze. The sorting of the HSC required excitation via UV laser and measuring the emission in the Indo-1 channel based on a 505 nm long pass filter. The sample loading port was set to 4 °C and 300 rpm. We used a 100 μm nozzle and a pressure of 20 psi. HBSS with calcium or magnesium was used as sheath fluid. The flow rate was set to 5000 events per second and the threshold was adjusted to 5000. The collection device was set to 4 °C. The collection tube was a 5 mL glass tube that contained 1 mL of Hank’s BSS without calcium or magnesium, 10 mM HEPES, and 20% of fetal bovine serum (FBS).

### Enzyme-linked immunosorbent assay

Isolated HSCs from normal and CDAHFD treated mice were cultured in six-well plates at a density of 4 × 10^5^ cells/well for 72 h. Supernatants were collected, and an ELISA for Ccl5 (Abcam, Cambridge, MA) was performed according to the manufacturer’s instructions. Results are expressed as Ccl5 secretion for 72 h.

### Neutralization assay

Neutralizing antibody to Ccl5 and normal goat immunoglobulin G control were procured (R&D Systems, Minneapolis, MN) and used (2 μg/mL) for the neutralization assay. Antibodies were incubated with conditioned media for 1 hour at 37 °C before applying it to primary hepatocytes.

### Statistical analysis

All the experiments were performed in triplicate and repeated at least two times. Data were expressed as mean +/− SD. Statistical analysis was performed using a Student t test for unpaired data to compare the values between the two groups and one-way analysis of variance among multiple groups. Differences in values were considered significant at P < 0.05.

## Electronic supplementary material

Supplementary Information
