# Author Correction: Hepatic stellate cells secrete Ccl5 to induce hepatocyte steatosis

**DOI:** 10.1038/s41598-020-67553-x

**Published:** 2020-06-19

**Authors:** Byeong-Moo Kim, Ahmed Maher Abdelfattah, Robin Vasan, Bryan C. Fuchs, Michael Y. Choi

**Affiliations:** 1 Department of Medicine, Gastrointestinal Unit, Massachusetts General Hospital and Harvard Medical School, Boston, MA 02114 USA; 2 Department of Surgery, Massachusetts General Hospital Cancer Center and Harvard Medical School, Boston, MA 02114 USA; 3 Division of Surgical Oncology, Massachusetts General Hospital Cancer Center and Harvard Medical School, Boston, MA 02114 USA; 4 Harvard Stem Cell Institute, Cambridge, MA 02138 USA

Correction to: *Scientific Reports* 10.1038/s41598-018-25699-9, published online 14 May 2018

This Article contains an error. The image used for the MVC panel in Figure 5B is incorrect. The correct Figure 5B appears below as Figure 1.

**Figure 1:**
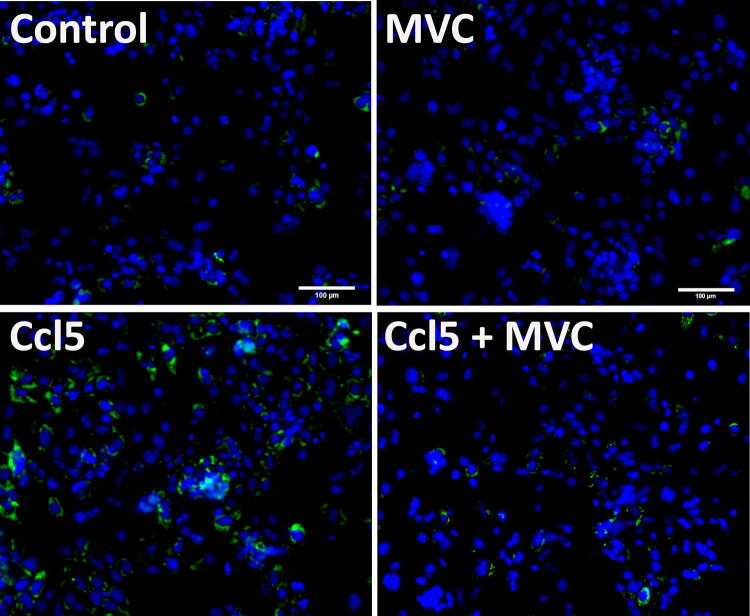
.

