# Supplementary material for: Hepatic stellate cells secrete Ccl5 to induce hepatocyte steatosis
Source: Sci Rep. 2018 May 14;8:7499. doi: 10.1038/s41598-018-25699-9 (PMC5951796; doi:10.1038/s41598-018-25699-9)
Supplement: Supplementary file 1 — Supplementary Information [file 41598_2018_25699_MOESM1_ESM.pdf]

Hepatic stellate cells secrete Ccl5 to induce hepatocyte steatosis

Byeong-Moo Kim, Ahmed Maher Abdelfattah, Robin Vasan, Bryan C. Fuchs, Michael Y. Choi*

Supplementary Information

Supplementary Figure 1. Cytokine array blot identified up-secretion of Ccl5 protein in the conditioned media of hepatic stellate cells isolated from CDAHFD induced steatohepatitis. CDAHFD, choline-deficient L-amino acid defined high fat diet; CM, conditioned media.

Supplementary Figure 2. Recombinant Ccl5 protein applied at various concentrations to mouse hepatocyte cell line AML12 demonstrated induction of steatosis observed with Bodipy staining.

Supplementary Figure 3. Full length Ccl5 was overexpressed in hepatic stellate cells with a vector that co-expresses green fluorescent protein. GFP, green fluorescent protein.

Supplementary Figure 4. Enzyme-linked immunosorbent assay demonstrated the up-secretion of Ccl5 by HSCs isolated from CDAHFD induced steatohepatitis. Incubating the Ccl5 containing media with a Ccl5 neutralizing antibody abrogated its signal. CDAHFD, choline-deficient L-amino acid defined high fat diet.

Supplementary Figure 5. Neutralizing Ccl5 in HSC conditioned media with a blocking antibody reduced steatosis in hepatocytes treated with the media. CDAHFD, choline-deficient L-amino acid defined high fat diet; CM, conditioned media.

Supplementary Figure 6. Hepatocyte culture lacks Ccl5 secreting HSC contamination demonstrated by Ccl5 immunofluorescence.

Supplementary table 1. Primer sequences.

Supplementary Figure 1.

Cytokine array blot identified up-secretion of Ccl5 protein in the conditioned media of hepatic stellate cells isolated from CDAHFD induced steatohepatitis. CDAHFD, choline-deficient L-amino acid defined high fat diet; CM, conditioned media.

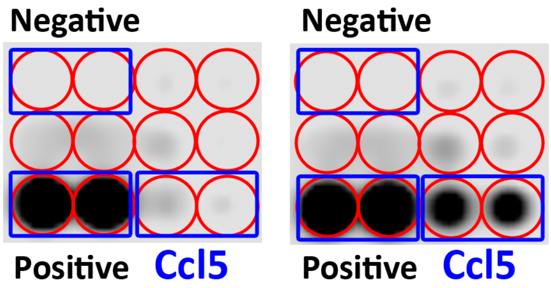

Supplementary Figure 2.
Recombinant Ccl5 protein applied at various concentrations to mouse hepatocyte cell line AML12 demonstrated induction of steatosis observed with Bodipy staining.

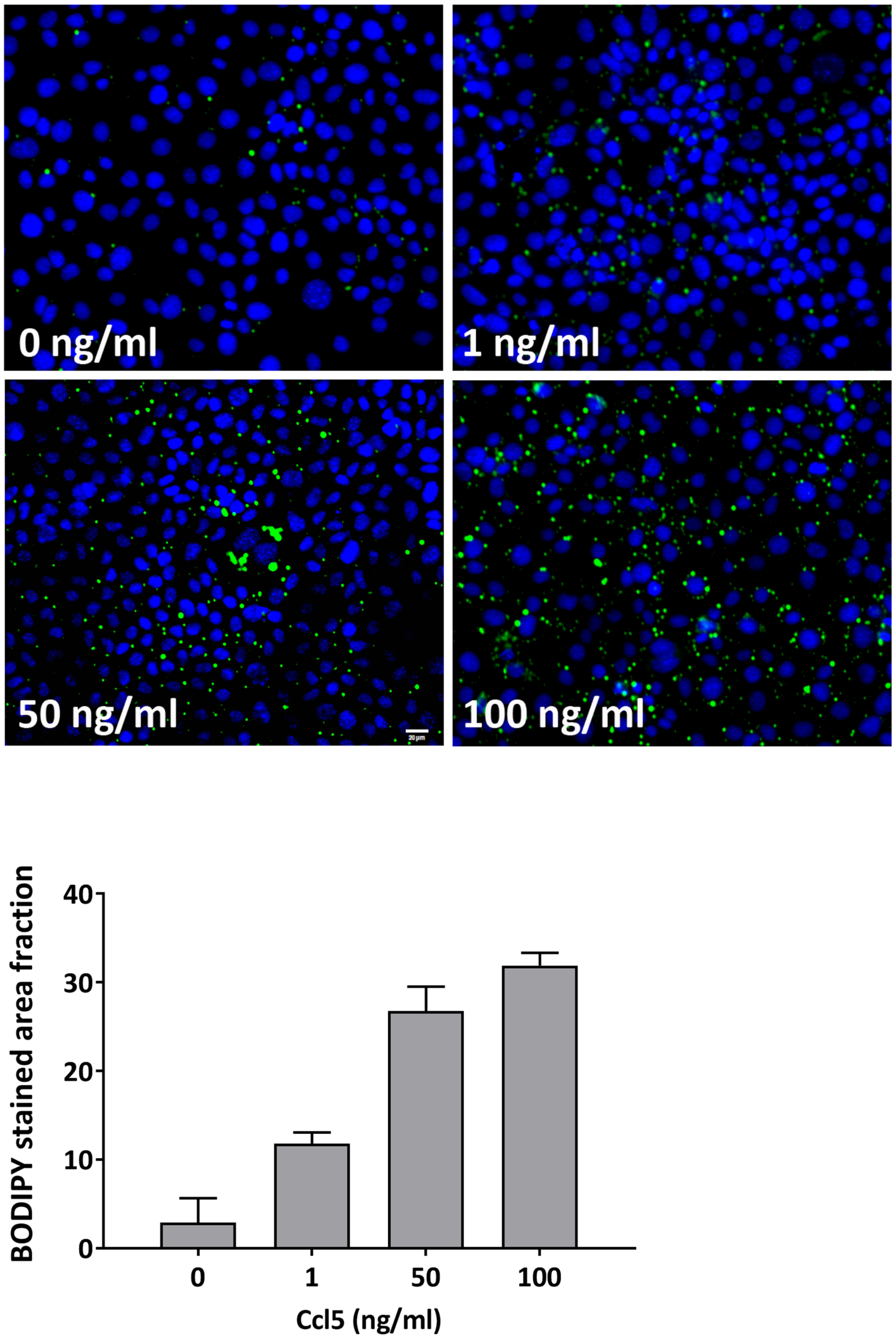

Supplementary Figure 3.
Full length Ccl5 was overexpressed in hepatic stellate cells with a vector that co-expresses green fluorescent protein. GFP, green fluorescent protein.

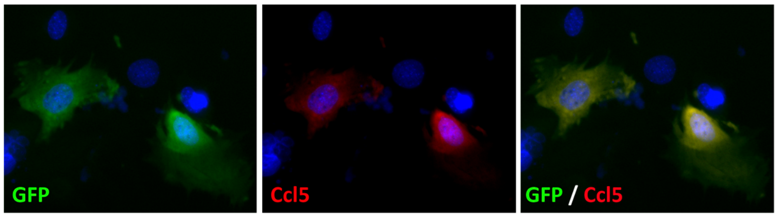

Supplementary Figure 4.
Enzyme-linked immunosorbent assay demonstrated the up-secretion of Ccl5 by HSCs isolated from CDAHFD induced steatohepatitis. Incubating the Ccl5 containing media with a Ccl5 neutralizing antibody abrogated its signal. CDAHFD, choline-deficient L-amino acid defined high fat diet.

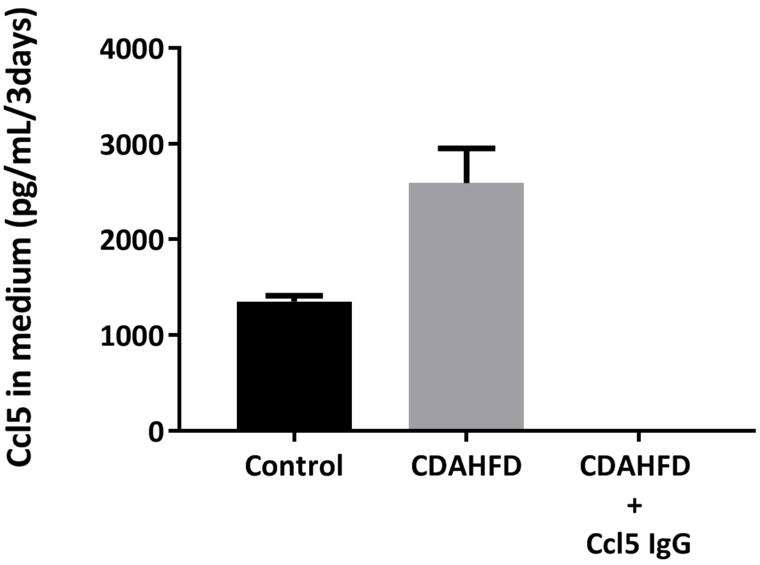

Supplementary Figure 5.
Neutralizing Ccl5 in HSC conditioned media with a blocking antibody reduced steatosis in hepatocytes treated with the media. CDAHFD, choline-deficient L-amino acid defined high fat diet; CM, conditioned media.

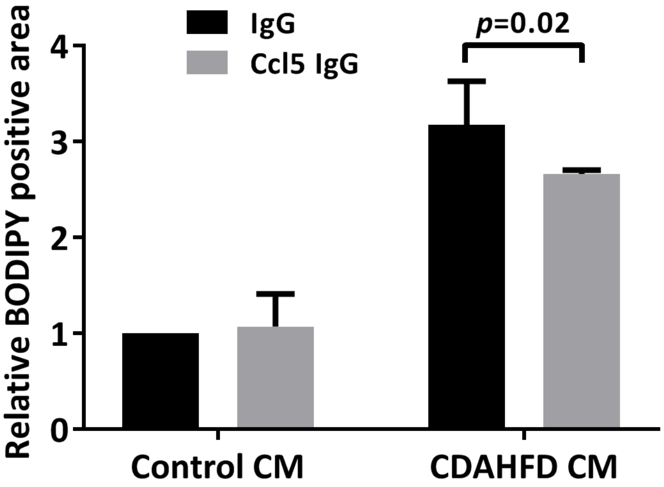

Supplementary Figure 6.
Hepatocyte culture lacks Ccl5 secreting HSC contamination demonstrated by Ccl5 immunofluorescence.

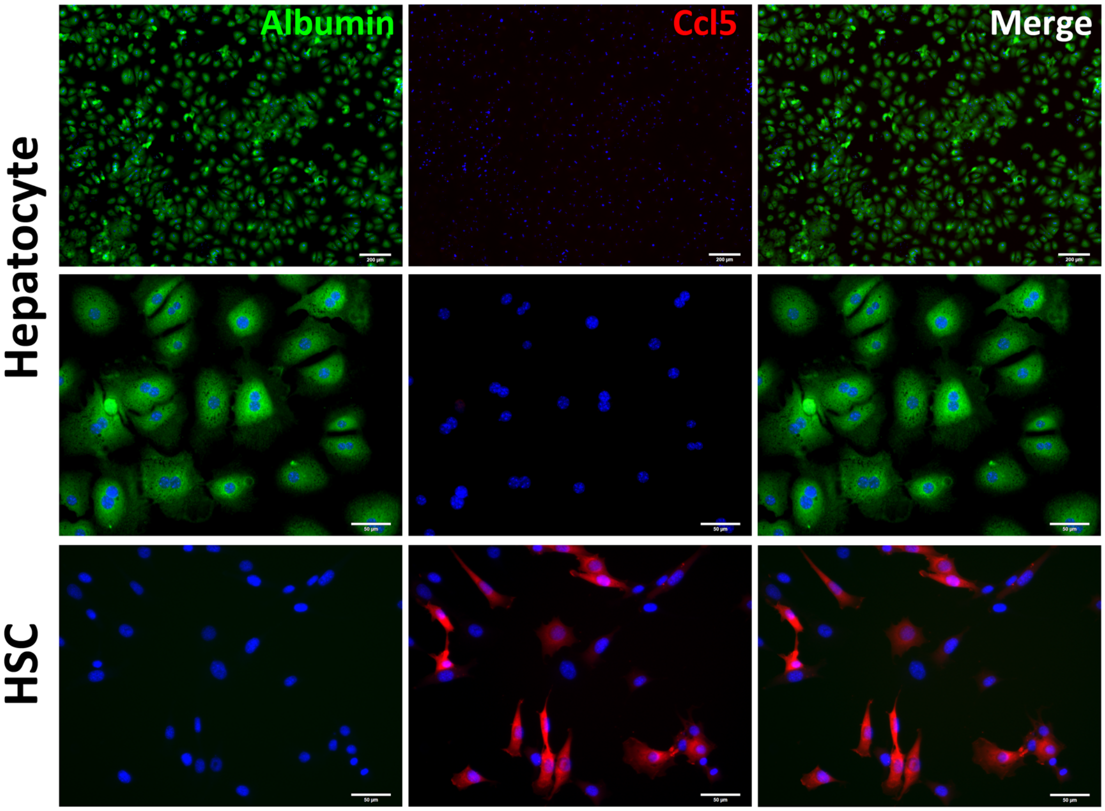

Supplementary Table S1. Primer sequences.

Genes	Forward	Reverse
Albumin	TGACTTTGCACAGTTCCTGGAT	AGGGTAGCCTGAGAAGGTTGTG
F4/80	CTTTGGCTATGGGCTTCCAGTC	GCAAGGAGGACAGAGTTTATCGTG
TNF-α	TCGAGTGACAAGCCTGTAGCCCACGTC	CTGCCCGGACTCCGCAAAGTCTAAGTAC
CD14	CTGCAGCAGTGGCTAAAGCCTGGACTCA	CCACTTGGGGCAGCTCATCTGGGCTAG
IL-1β	CTTTGAAGTTGACGGACCC	TGAGTGATACTGCCTGCCTG
IL-6	ACAACCACGGCCTTCCCTACTT	CACGATTTCCCAGAGAACATGTG
MCP-1	CAGGTCCCTGTCATGCTTCT	CAGGTCCCTGTCATGCTTCT
Ccl3	TCTCAGCGCCATATGGAGCT	TTCCGGCTGTAGGAGAAGCA
Ccl4	CCGAGCAACACCATGAAGC	CCATTGGTGCTGAGAACCCT
Ccl5	GCTGCTTTGCCTACCTCTCC	TCGAGTGACAAACACGACTGC
Ccl8	CCAGATAAGGCTCCAGTCACCT	GGCACTGGATATTGTTGATTCTCTC
Ccl7	GCAGAGAAGCAAGGCCAGCACA	AGCAGGCACAGAAGCGTGGC
Ccr1	GGCTACAGGTACGGTGAGTGAAC	CTGGCCATTGTCCATGCTGTG
Ccr3	AAGCTTTGAGACCACACCCTATG	GACCCCAGCTCTTTGATTCTGA
Ccr5	CACAGCATGGACAATAGCCAAGTACC	GCCATCTCTGACCTGCTCTTCC
Col1a1	TGTGTGCGATGACGTGCAAT	GGGTCCCTCGACTCCTACA
Acta2	ATTGTGCTGGACTCTGGAGATGGT	TGATGTCACGGACAATCTCACGCT
Gapdh	TTCACCACCATGGAGAAGGC	CCCTTTTGGCTCCACCCT
